# 200 Years of Psychosomatic Medicine—And Still More Timely Than Ever

**DOI:** 10.3389/fpsyt.2018.00674

**Published:** 2018-12-06

**Authors:** Stephan Zipfel

**Affiliations:** Department of Psychosomatic Medicine and Psychotherapy, University Hospital Tübingen, Tübingen, Germany

**Keywords:** psychosomatic medicine, placebo, psycho-oncology, mind and body, psychotherapy, refugee, gut microbiome

Heinroth (1) introduced the term “psychosomatic” in his famous textbook of “Disturbances of Mental Life” into the medical literature. In addition Heinroth's major importance to the field of Psychosomatic Medicine is also due to his early holistic and anthropological approach to Medicine and Psychiatry. To date psychosomatic medicine has developed rapidly over the last decades, combining its two traditions of integrated psychosomatics in internal medicine and focusing on psychotherapeutic/psychiatric methods in many clinical fields (2).

Our section of “Psychosomatic Medicine” in Frontiers in Psychiatry gives this timely development a highly visible platform. Over the last year our Research Topic on “Nutritional Psychiatry” is an excellent example to demonstrate how cutting edge science help to understand how the brain-gut interactions work (3) and how the gut microbiome is linked to neurodevelopment and depression (4). Currently, a team of editors from very experienced placebo researchers gives us an insight into the fascinating mechanisms of placebo response in experimental settings as well as in clinical applications.

Psychosomatic medicine also has a social and political side—which is why we have also focussed on the challenge of refugee mental health, a highly challenging field. In a current Research Topic we have dedicated ourselves particularly to the special strains of extremely traumatized groups like the Yezidi people. Nadia Murad, a young woman of this ethnic group was awarded the Nobel Peace Prize this year for her courageous appearance. Questions also arise as to how high the burden is for the professional supporters (5) and also which new media approaches can be used to support the group of traumatized refugees (6). In a recent systematic review we demonstrated (7) that chronic pain in patients with posttraumatic stress disorder (PTSD) is a frequent symptom and a complicating factor in the treatment.

Psychotherapy is the primary therapy method for patients with psychosomatic disorders. Therefore, our section also wants to be a place where studies on new and innovative psychotherapeutic procedures are reported. But our journal should also be a place where the various psychotherapeutic methods can be openly debated (8).

And if we look into the year 2019, then we're going to highlight together with an experienced international research team the central aspect of body image in biopsychosocial medicine in a further research topic. Clinically highly relevant fields such as psycho-oncology will also be given a broader focus in the upcoming year. Thus, psychosomatic medicine continues to grow and thrive in our journal and bears fruit for a better understanding of the interaction between mind and body.

## Author Contributions

The author confirms being the sole contributor of this work and has approved it for publication.

### Conflict of Interest Statement

The author declares that the research was conducted in the absence of any commercial or financial relationships that could be construed as a potential conflict of interest.
